# The early posterior cortex pixel value ratio: a novel reliable indicator for distraction osteogenesis

**DOI:** 10.3389/fsurg.2023.1280332

**Published:** 2023-10-30

**Authors:** Ze Liu, Qi Liu, Min Wang, Chenhan Zhou, Hongbin Guo, Jieyu Liang, Yi Zhang

**Affiliations:** ^1^ Department of Orthopaedics, Xiangya Hospital, Central South University, Changsha, China; ^2^ National Clinical Research Center for Geriatric Disorders, Xiangya Hospital, Central South University, Changsha, China; ^3^ Department of Orthopaedics, Seventh Clinical Medical College, Beijing Jishuitan Hospital, Capital Medical University, Beijing, China; ^4^ Department of Endocrinology, Xiangya Hospital, Central South University, Changsha, China

**Keywords:** bone union and nonunion, distraction osteogenesis, external fixator, pixel value ratio, bone regeneration index, healing index, lengthening index, external fixator index

## Abstract

**Aims:**

We aimed to explore the associations of the early PVR in four cortices with Healing Index (HI), Lengthening Index (LI), and External Fixator Index (EFI) in the bone union and non-union groups.

**Methods:**

A total of 52 patients, including 39 bone union and 13 bone non-union subjects, were recruited in this study. The general characteristics and PVR in four cortices in each group were explored. Afterward, the early PVR in four cortices, including medial, lateral, anterior, and posterior sides, were compared. Finally, the associations of the early PVR in four cortices with HI, LI, and EFI were also investigated.

**Results:**

The general characteristics of these patients were consistent, except for HI (31.54 ± 12.24 vs. 45.08 ± 27.10, *P* = 0.018) and EFI (57.63 ± 18.15 vs. 71.29 ± 24.60, *P* = 0.046). The growth of regenerated callus was asymmetrical in the bone union group (the posterior PVR seems to grow faster), whereas no statistical difference was obtained in the bone non-union group. Furthermore, the posterior PVR in the bone union group was significantly higher than that in the bone non-union group (the first month: 0.96 ± 0.17 vs. 0.86 ± 0.06, *p* = 0.047; the second month: 0.98 ± 0.14 vs. 0.89 ± 0.09, *p* = 0.041; the third month: 1.00 ± 0.12 vs. 0.92 ± 0.09, *p* = 0.039). Most importantly, the posterior PVR was inversely associated with HI, LI, and EFI (the first month: *r* = −0.343, *p* = 0.041; *r* = −0.346, *p* = 0.042; *r* = −0.352, *p* = 0.041; the second month: *r* = −0.459, *p* = 0.004; *r* = −0.277, *p* = 0.101; *r* = −0.511, *p* = 0.002; the third month: *r* = −0.479, *p* = 0.003; *r* = −0.398, *p* = 0.018; *r* = −0.551, *p* = 0.001) in the bone union group, respectively. However, this finding was lost in the bone non-union group.

**Conclusion:**

The early posterior cortex PVR seems to grow faster than the medial, lateral, and anterior sides in the bone union group, which represents an asymmetrical development pattern. Moreover, the posterior cortex PVR was negatively associated with HI, LI, and EFI, respectively. The posterior cortex PVR may be a novel and reliable detection index in the process of DO.

## 1. Introduction

Distraction osteogenesis (DO) is a method that promotes bone tissue regeneration and growth by continuous and slow traction for bone lengthening (extending peripheral nerves, blood vessels, muscles, and skin) (1, 2). Osteotomy is performed on the bone that needs to be lengthened initially. After a short latency period, a distractor is utilized to distract the bone at an appropriate speed/frequency to spontaneously promote bone regeneration in the distraction gap. This period is referred to as the distraction period. Next, a long period of consolidation is needed to achieve new bone mineralization and remodeling (3). The llizarov technique used in DO dates back to 1951 and is widely utilized nowadays to treat bone defects, limb unequal lengths, and deformities (4, 5). However, DO requires regular monitoring of callus growth due to the long treatment time, possible skin scar, psychological and life burden, pain syndrome, joint stiffness, pin tract infection, and bone non-union (6). Among them, bone non-union is a serious clinical issue, which prolongs the treatment course and increases the difficulty in bone extension (7). Paley et al. have found that both technical factors (traumatic corticotomy, instability, and rapid traction) and patient factors (infection, malnutrition, and metabolism) may contribute to the DO-related non-union (8). However, the current effective prediction methods of bone non-union are quite limited. Several methods were used to monitor DO, such as quantitative computed tomography (QCT), dual-energy x-ray absorptiometry (DXA), standard radiography (x-ray), ultrasound, biomechanical evaluation, and biochemical marker. Among them, x-ray is the most convenient and affordable approach (9, 10). Additionally, the pixel value ratio (PVR) can be measured by digital x-rays, which have been shown to correlate well with bone mineral density (BMD) of regenerated bone. Therefore, PVR is a quantitative indicator of mineralization in DO healing (11, 12). As far as we know, the major previous studies are related to the later stage for the timing of removing the external fixator (13–15), and less attention is paid to the early PVR. Our recent studies found the associations of early PVR with healing index, (HI: the time to complete consolidation (days) divided by the length obtained (cm)), lengthening index (LI: the number of months required to achieve 1 cm lengthening), and external fixator index (EFI: dividing the using period of external fixator (days) by the distracted length of the bone (cm)) (16). However, E. Vulcano et al. showed the uneven PVR in four cortices of the callus during the late weight-bearing period of intramedullary extension (lateral: 0.84, medial: 0.89, anterior: 0.92, posterior: 0.98). It is not clear whether this condition exists in the early stage of DO and which cortex is related to HI, LI, and EFI (14). Furthermore, our other previous study found that the PVR growth pattern in bone union differed from that in bone non-union (17). Therefore, the intention of this study was to compare the early measurements of the PVR in two cohorts of patients: those who had uncomplicated bone lengthening and those who developed failure of the regenerate bone to form properly. This study aims to: (1) compare the early PVR in four cortices of the bone union and non-union groups; (2) clarify the different growth patterns of early PVR in four cortices in bone union and non-union groups; (3) identify the associations of early PVR in four cortices with HI, LI, and EFI, respectively.

## 2. Materials and methods

### 2.1. Study design and patients

This study was approved by the Ethics Committee of Xiangya Hospital of Central South University. We retrospectively analyzed the clinical data and images of patients who underwent bone lengthening surgery at Xiangya Hospital of Central South University from January 2010 to April 2023. The inclusion criteria were: (1) The lower extremities were lengthened using the Ilizarov method; (2) Patients with bone union and non-union during DO. The diagnostic standards of bone union and bone non-union: bone union indicated the ones where the external fixator was removed successfully, whereas bone non-union represented the ones where bridging callus did not appear even after 9 months (an absence of bridging callus for at least three out of four cortices on plain radiographs) that needs autogenous bone transplantation (18–20). (3) The patients who had primary surgery. The exclusion criteria were: (1) The patients who could not complete the full course of bone lengthening therapy; (2) The patients who suffered from bone diseases that affected bone healing (e.g., osteomyelitis); (3) The patients who were lost to follow-up.

### 2.2. Surgical methods

The surgery involved in this study was performed by senior surgeons in Xiangya Hospital of Central South University. A tibial or femoral osteotomy was performed and a ring or unilateral external fixation fixator was then placed. The bone lengthening was initiated 1 week after the operation (the extension rate is 0.75 mm/day for adults and 1 mm/day for adolescents), and the growth of callus was monitored monthly (detailed distraction osteogenesis parameters are shown in Table 1). The standard for removing external fixtures was listed as follows: (1) At least three of the four cortices of the extension are fully developed according to anteroposterior and lateral x-ray photographs; (2) The fixation time generally conforms to the average stretch index (1 month of consolidation time is required for every 1 cm of extended callus); (3) There is no abnormal feeling of complete load-bearing after loosening the nut (16, 21).

**Table 1:** The basic distraction osteogenesis parameters between bone union and non-union.

	Bone union	Bone non-union	*P*-value
Distraction rate (mm/day)	0.75	0.75	
Distraction rhythm (times/day)	3	3	
Distraction length (cm)	5.61 ± 3.32	7.73 ± 3.97	*P* = 0.066
Distraction time (day)	275.17 ± 146.83	529.42 ± 350.68	***P* = 0.001**

The bold values indicate the statistical significant *P* value (<0.05).

### 2.3. Pixel value ratio measurement based on x-ray

**Figure 1:**
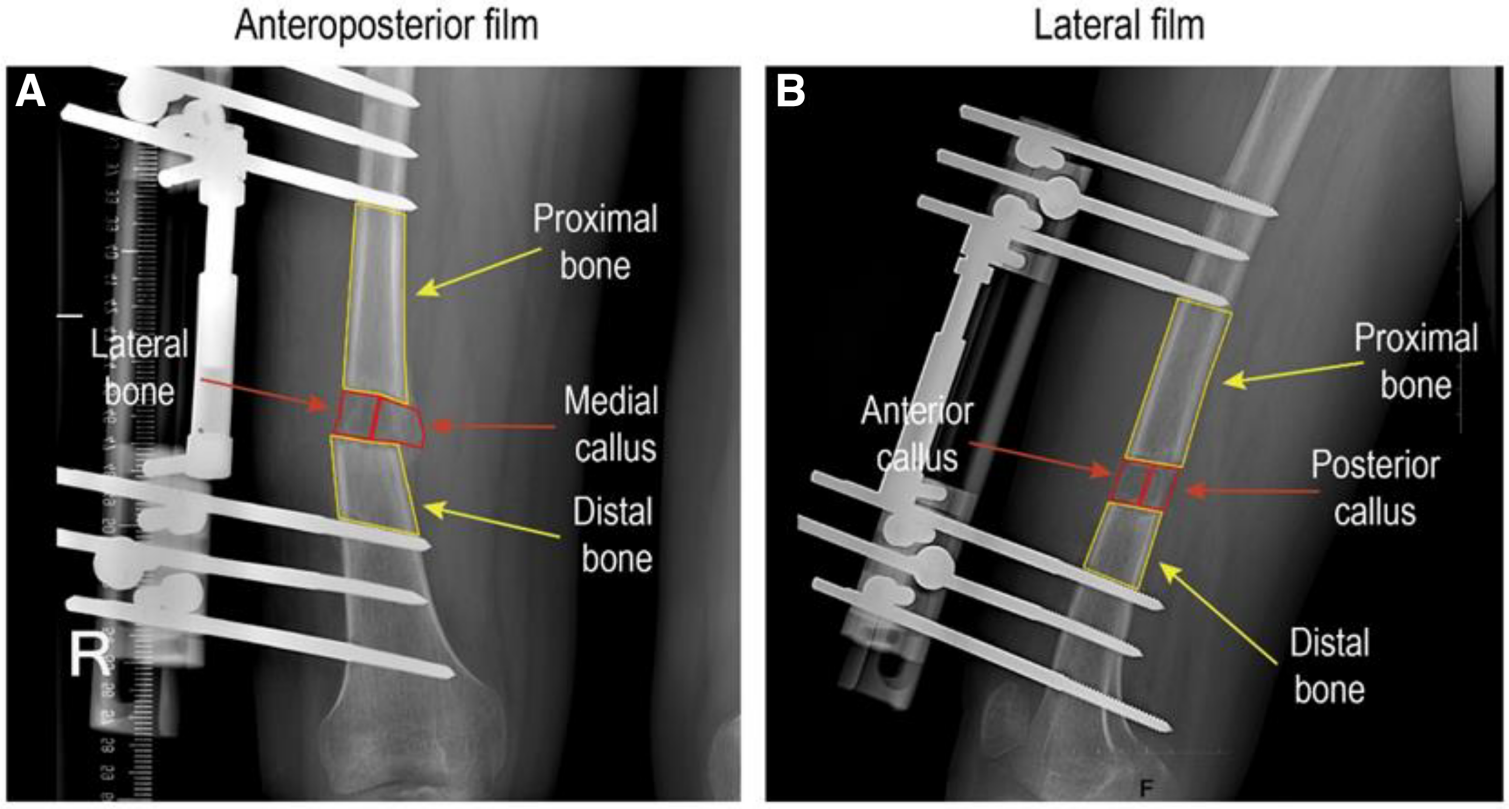
The assesment nof lateral, medial, anterior and posterior callus pixel value ratio.

The Picture Archiving and Communication System (PACS) of Xiangya Hospital of Central South University was utilized to measure x-rays of recruited patients, including anterior callus, posterior callus, medial callus, lateral callus, proximal bone, and distal bone (Figure 1). All data were tested by the same technician/equipment and evaluated by two senior orthopedic surgeons independently. The individual data with different opinions were recalled and fully discussed. In the measurement process, the interference of the metal frame was carefully avoided to ensure the accuracy of the data analysis. The higher PVR indicated that the callus was closer to a normal bone, whereas the lower PVR represented the immature regenerated callus (22). The formula is as follows (12, 14):

$$\mathrm{PVR}=\frac{\mathrm{Regenerated}\;\mathrm{medial}/\mathrm{lateral}/\mathrm{posterior}/\mathrm{anterior}\;\mathrm{callus}\;\mathrm{pixel}\;\mathrm{value}}{(\mathrm{Distal}\;\mathrm{normal}\;\mathrm{bone}\;\mathrm{pixel}\;\mathrm{value}+\mathrm{Proximal}\;\mathrm{normal}\;\mathrm{bone}\;\mathrm{pixel}\;\mathrm{value})÷2}$$

### 2.4. The general characteristics of patients and the PVR in four cortices analyses, and their associations with the healing index, lengthening index, and external fixator index

A total of 52 patients were recruited in this study (including 39 bone union and 13 bone non-union subjects). The general characteristics of the patients including sex, age, body mass index (BMI), osteotomy location, external fixator type, healing index (HI), lengthening index (LI), and external fixator index (EFI) were collected and analyzed. Based on the anteroposterior and lateral x-rays, the callus was divided into four sides: anterior, posterior, medial, and lateral. The PVR in four cortices of the callus was collected months after osteotomy, which were further analyzed and compared. Moreover, the anterior, posterior, medial, and lateral cortical PVRs of the bone union and non-union were also analyzed and compared. HI was calculated by dividing the time of tricortical formation (days) by the length of extension (centimeters) (23). LI referred to the time it took to extend one centimeter in months (24). EFI was the time spent wearing the external fixation frame (days) divided by the extension length (cm) (25). All three indices were valid indicators, which reflected the final clinical outcome of DO well. The associations of early PVR in four cortices with the HI, LI, and EFI were investigated in our study.

### 2.5. Statistical analysis

All analyses were performed using SPSS version 26.0 software. The differences in general characteristics between the bone union and non-union groups were analyzed by multivariate analysis of variance. The analysis of variance was used to analyze the differences in four cortices PVR within the bone union and non-union groups. The correlation between PVR in four cortices of callus and HI/LI/EFI in bone union and non-union groups was performed by Pearson correlation analysis. *P* < 0.05 was considered statistically significant.

## 3. Results

### 3.1. The general characteristics of the included subjects

The general characteristics of the patients are shown in Table 2. Most of the basic characteristics of the bone union and bone non-union groups were similar, except for two indices (HI: 31.54 ± 12.24 vs. 45.08 ± 27.10, *P* = 0.018; EFI: 57.63 ± 18.15 vs. 71.29 ± 24.60, *P* = 0.046).

**Table 2:** The basic information between bone union and non-union.

		Bone union	Bone non-union	*P*-value
Sex	Man	51.3%	46.2%	*P* = 0.173
	Woman	48.7%	53.8%	
Age (years)		27.56 ± 12.78	28 ± 12.53	*P* = 0.914
BMI		22.02 ± 4.76	20.75 ± 4.63	*P* = 0.460
HI		31.54 ± 12.24	45.08 ± 27.10	***P* = 0.018**
LI		0.77 ± 0.43	0.78 ± 0.26	*P* = 0.956
EFI		57.63 ± 18.15	71.29 ± 24.60	***P* = 0.046**
External fixation type	Unilateral	23.1%	38.5%	*P* = 0.269
	Ring	76.9%	61.5%	
Site of osteotomy	Tibia	64.1%	30.8%	*P* = 0.203
	Femur	35.9%	69.2%	

The bold values indicate the statistical significant *P* value (<0.05).

### 3.2. The asymmetric growth of early callus PVR in bone union

The growth of early callus PVR in bone union was asymmetrical. The anterior, posterior, medial, and lateral PVR of the first, second, and third months were (0.80 ± 0.18, 0.96 ± 0.17, 0.88 ± 0.16, 0.89 ± 0.15, and *P* = 0), (0.82 ± 0.17, 0.98 ± 0.14, 0.90 ± 0.16, 0.90 ± 0.17, and *P* = 0.01), (0.85 ± 0.13, 1.00 ± 0.12, 0.92 ± 0.12, 0.95 ± 0.17, and *P* = 0), respectively. However, no significant statistical differences were obtained in the non-union group (Table 3).

**Table 3:** The PVR in four cortices in the first three months after osteotomy.

Group	PVR	The first month		The second month		The third month	
Bone union	Anterior cortex	0.80 ± 0.18	***P *< 0.0001**	0.82 ± 0.17	***P *< 0.0001**	0.85 ± 0.13	***P *< 0.0001**
	Posterior cortex	0.96 ± 0.17		0.98 ± 0.14		1.00 ± 0.12	
	Medial cortex	0.88 ± 0.16		0.90 ± 0.16		0.92 ± 0.12	
	Lateral cortex	0.89 ± 0.15		0.90 ± 0.17		0.95 ± 0.17	
Bone non-union	Anterior cortex	0.78 ± 0.09	*P *= 0.172	0.81 ± 0.09	*P *= 0.085	0.81 ± 0.08	***P *= 0.02**
	Posterior cortex	0.86 ± 0.06		0.89 ± 0.09		0.92 ± 0.09	
	Medial cortex	0.83 ± 0.11		0.87 ± 0.09		0.90 ± 0.07	
	Lateral cortex	0.85 ± 0.12		0.88 ± 0.08		0.91 ± 0.05	

The bold values indicate the statistical significant *P* value (<0.05).

### 3.3. The early posterior PVR in bone union was significantly higher than that in bone non-union

The early posterior PVR in bone union was significantly higher than that in bone non-union: 0.96 ± 0.17 vs. 0.86 ± 0.06, *p* = 0.047 (first month); 0.98 ± 0.14 vs. 0.89 ± 0.09, *p* = 0.041 (second month); 1.00 ± 0.12 vs. 0.92 ± 0.09, *p* = 0.039 (third month). However, no significant statistical differences were obtained in anterior, medial, and lateral PVR (Table 4).

**Table 4:** The comparison of PVR in four cortices in the first three months for bone union and non-union.

		Bone union	Bone non-union	*P-*value
The first month	Anterior cortex PVR	0.80 ± 0.18	0.78 ± 0.09	0.773
	Posterior cortex PVR	0.96 ± 0.17	0.86 ± 0.06	**0**.**047**
	Medial cortex PVR	0.88 ± 0.16	0.83 ± 0.11	0.375
	Lateral cortex PVR	0.89 ± 0.15	0.85 ± 0.12	0.425
The second month	Anterior cortex PVR	0.82 ± 0.17	0.81 ± 0.09	0.749
	Posterior cortex PVR	0.98 ± 0.14	0.89 ± 0.09	**0**.**041**
	Medial cortex PVR	0.90 ± 0.16	0.87 ± 0.09	0.500
	Lateral cortex PVR	0.90 ± 0.17	0.88 ± 0.08	0.619
The third month	Anterior cortex PVR	0.85 ± 0.13	0.81 ± 0.08	0.305
	Posterior cortex PVR	1.00 ± 0.12	0.92 ± 0.09	**0**.**039**
	Medial cortex PVR	0.92 ± 0.12	0.90 ± 0.07	0.481
	Lateral cortex PVR	0.95 ± 0.17	0.91 ± 0.05	0.319

The bold values indicate the statistical significant *P* value (<0.05).

### 3.4. Associations of the healing index, lengthening index, and external fixator index with the early pixel value ratio in four cortices of the callus

The early posterior PVR was inversely associated with HI in the bone union group: *r* = −0.343, *p* = 0.041 (first month); *r* = −0.459, *p* = 0.004 (second month); *r* = −0.479, *p* = 0.003 (third month). In addition, the early posterior PVR of the bone union group was inversely associated with LI in the bone union group: *r* = −0.346, *p* = 0.042 (first month); *r* = −0.277, *p* = 0.101 (second month); *r* = −0.398, *p* = 0.018 (third month). With regard to EFI, the early posterior PVR was also inversely associated with EFI in the bone union group: *r* = −0.352, *p* = 0.041 (first month); *r* = −0.511, *p* = 0.002 (second month); *r* = −0.551, *p* = 0.001 (third month). However, interestingly, such a relationship was lost on the other three sides (Table 5). Moreover, the negative associations of posterior PVR with HI, LI, and EFI were not obtained in bone non-union (Table 6).

**Table 5:** The associations of early PVR in four cortices with the healing index, lengthening index, and external fixator index in bone union.

Bone union	HI	LI	EFI
The first month			
Anterior cortex PVR	*r* = −0.296, *p* = 0.080	*r* = −0.181, *p* = 0.297	r = −0.220, *p* = 0.212
Posterior cortex PVR	***r* = −0.343, *p* = 0.041**	***r* = −0.346, *p* = 0.042**	**r = −0.352, *p* = 0.041**
Medial cortex PVR	*r* = −0.099, *p* = 0.565	***r* = −0.345, *p* = 0.042**	*r* = −0.032, *p* = 0.855
Lateral cortex PVR	*r* = −0.274, *p* = 0.105	***r* = −0.393, *p* = 0.018**	*r* = −0.190, *p* = 0.281
The second month			
Anterior cortex PVR	*r* = −0.012, *p* = 0.946	*r* = 0.219, *p* = 0.200	*r* = 0.033, *p* = 0.851
Posterior cortex PVR	***r* = −0.459, *p* = 0.004**	*r* = −0.277, *p* = 0.101	***r* = −0.511, *p* = 0.002**
Medial cortex PVR	*r* = 0.038, *p* = 0.826	*r* = 0.177, *p* = 0.302	*r* = 0.157, *p* = 0.367
Lateral cortex PVR	*r* = 0.101, *p* = 0.552	*r* = 0.209, *p* = 0.221	*r* = 0.261, *p* = 0.130
The third month			
Anterior cortex PVR	*r* = 0.094, *p* = 0.586	*r* = 0.223, *p* = 0.197	*r* = 0.077, *p* = 0.666
Posterior cortex PVR	***r* = −0.479, *p* = 0.003**	***r* = −0.398, *p* = 0.018**	***r* = −0.551, *p* = 0.001**
Medial cortex PVR	*r* = −0.100, *p* = 0.561	*r* = 0.103, *p* = 0.558	*r* = −0.324, *p* = 0.061
Lateral cortex PVR	*r* = −0.160, *p* = 0.350	*r* = −0.002, *p* = 0.989	*r* = −0.268, *p* = 0.126

The bold values indicate the statistical significant *P* value (<0.05).

**Table 6:** The associations of early PVR in four cortices with the healing index, lengthening index, and external fixator index in bone non-union.

Bone non-union	HI	LI	EFI
The first month			
Anterior cortex PVR	*r* = −0.225, *p* = 0.461	*r* = 0.177, *p* = 0.563	*r* = −0.118, *p* = 0.716
Posterior cortex PVR	*r* = 0.120, *p* = 0.696	*r* = 0.524, *p* = 0.066	*r* = 0.097, *p* = 0.763
Medial cortex PVR	*r* = −0.127, *p* = 0.678	*r* = 0.166, *p* = 0.588	*r* = 0.046, *p* = 0.887
Lateral cortex PVR	*r* = −0.124, *p* = 0.686	*r* = 0.097, *p* = 0.751	*r* = −0.044, *p* = 0.892
The second month			
Anterior cortex PVR	*r* = −0.488, *p* = 0.091	*r* = −0.036, *p* = 0.908	*r* = −0.458, *p* = 0.134
Posterior cortex PVR	*r *= −0.035, *p* = 0.910	*r* = −0.306, *p* = 0.309	*r* = −0.049, *p* = 0.879
Medial cortex PVR	*r* = −0.435, *p* = 0.138	*r* = 0.082, *p* = 0.791	*r* = −0.210, *p* = 0.512
Lateral cortex PVR	*r* = −0.335, *p* = 0.264	*r* = 0.049, *p* = 0.873	*r* = −0.152, *p* = 0.637
The third month			
Anterior cortex PVR	*r* = 0.048, *p* = 0.873	*r* = −0.235, *p* = 0.439	*r* = 0.396, *p* = 0.203
Posterior cortex PVR	*r* = −0.173, *p* = 0.571	*r* = −0.341, *p* = 0.254	*r* = −0.391, *p* = 0.209
Medial cortex PVR	***r* = −0.672, *p* = 0.012**	*r* = 0.019, *p* = 0.950	*r* = −0.427, *p* = 0.167
Lateral cortex PVR	*r* = −0.282, *p* = 0.351	*r* = 0.188, *p* = 0.540	*r* = 0.033, *p* = 0.918

The bold values indicate the statistical significant *P* value (<0.05).

## 4. Discussion

Previous studies have investigated the late PVR to consider the timing for external fixator removal. E. Vulcano et al. declared that an overall mean PVR of 0.90 was representative of clinical bone healing (14). A. Bafor et al. found that the participants started weight-bearing with no adverse effects when three of four cortices had a PVR of at least 0.93 (12). Moreover, L. Zhao et al. concluded that the PVR criteria for partial and full weight-bearing were the two/three cortical PVR close to one, respectively (22). Besides, the discovery of three or four consecutive cortices at least 2 mm thick on anteroposterior and lateral radiographs is a common criterion for removing external fixation frames after DO (26, 27). However, the above evidence was related to the intramedullary lengthening, and the early PVR in four cortices was also ignored. Therefore, this study was employed to address these issues.

Our previous study has shown that the early PVR was moderately negatively associated with HI and LI, which suggests that the early PVR can effectively reflect the clinical outcome of DO (16). However, the four cortices were taken as a whole. Considering the different rates of PVR growth in the four cortices, it is not clear which side of PVR matters a lot (12, 14, 22). In addition, we also found that the PVR growth pattern between bone union and non-union was quite different (17). Therefore, the present study aimed to investigate the growth of early PVR in four cortices for the bone union and non-union groups and analyzed their relationship with HI, LI, and EFI, respectively. Our study found that the early posterior cortex PVR in the bone union group grew faster and was negatively associated with HI, LI, and EFI in bone union, rather than in bone non-union. This result is consistent with E. Vulcano et al.'s study (the four cortical PVRs were: lateral 0.84, medial 0.89, anterior 0.92, and posterior 0.98) (14). It was speculated that the posterior cortex had less periosteum (necessary for bone tissue growth) and soft tissue destruction during osteotomy. The periosteum has three layers of structure: the superficial fibrous layer, the middle vascular undifferentiated area, and the inner cambium layer, which is adjacent to the outer surface of the bone with decent osteogenic ability (28, 29). Importantly, the callus formation was significantly impaired (failed bone lengthening) after periosteum removal (30). Furthermore, K. Nakahara et al.'s study proved that the periosteum plays an indispensable indirect role in the process of DO (31). The above evidence seems to support the asymmetric growth pattern of early callus PVR in bone union.

## 5. Advantages and limitations

The advantages of this study are as follows: (1) this is the first extramedullary extension study to monitor early PVR in four cortices during DO; (2) our study has specified the early posterior cortex PVR as a potential novel reliable indicator for DO. The limitations of our study should also be acknowledged: (1) several issues cannot be addressed due to the nature of the retrospective study design; (2) interference from metal fixtures during PVR measurement may have a slight impact on PVR evaluation; (3) the number of recruited subjects is relatively small, which may inevitably influence our results.

## 6. Conclusion

The early posterior cortex PVR seems to grow faster than the medial, lateral, and anterior sides in the bone union group, which represents an asymmetrical development pattern. Moreover, the posterior cortex PVR was negatively associated with HI, LI, and EFI, respectively. The posterior cortex PVR may be a novel and reliable detection index in the process of DO. Our results suggest that the early posterior cortex PVR should be noticed in clinical practice, which provides important information for accurate monitoring of callus in subjects with DO. However, due to the small sample size and retrospective design of this study, further large-scale prospective studies are needed to support the issues concerned.

## Data Availability

The original contributions presented in the study are included in the article/Supplementary Material, further inquiries can be directed to the corresponding authors.
